# Social determinants of ambulatory care sensitive conditions: a qualitative meta-synthesis based on patient perspectives

**DOI:** 10.3389/fpubh.2023.1147732

**Published:** 2023-05-09

**Authors:** Hsueh-Fen Chen, Hung-Ru Lin

**Affiliations:** ^1^Department of Healthcare Administration and Medical Informatics, College of Health Sciences, Kaohsiung Medical University, Kaohsiung City, Taiwan; ^2^Department of Medical Research, Kaohsiung Medical University Hospital, Kaohsiung City, Taiwan; ^3^Center for Big Data Research, Kaohsiung Medical University, Kaohsiung City, Taiwan; ^4^School of Nursing, National Taipei University of Nursing and Health Sciences, Taipei City, Taiwan

**Keywords:** social determinants, ambulatory care-sensitive conditions, meta-synthesis, qualitative studies, disease management, patient perspectives

## Abstract

**Background:**

Hospitalizations or emergency department (ED) visits due to ambulatory care-sensitive conditions (ACSC) are preventable but cost billions in modern countries. The objective of the study is to use a meta-synthesis approach based on patients' narratives from qualitative studies to reveal why individuals are at risk of ACSC hospitalizations or ED visits.

**Methods:**

PubMed, Embase, Cochrane Library, and Web of Science databases were utilized to identify qualified qualitative studies. The Preferred Reporting Items for Systematic Review and Meta-Analysis were used for reporting the review. The thematic synthesis was used to analyze the data.

**Results:**

Among 324 qualified studies, nine qualitative studies comprising 167 unique individual patients were selected based on the inclusion/exclusion criteria. Through the meta-synthesis, we identified the core theme, four major themes, and the corresponding subthemes. Poor disease management, the core theme, turns individuals at risk of ACSC hospitalizations or ED visits. The four major themes contribute to poor disease management, including difficulties in approaching health services, non-compliance with medications, difficulties in managing the disease at home, and poor relationships with providers. Each major theme comprised 2–4 subthemes. The most cited subthemes are relative to upstream social determinants, such as financial constraints, inaccessible health care, low health literacy, psychosocial or cognitive constraints.

**Conclusion:**

Without addressing upstream social determinants, socially vulnerable patients are unlikely to manage their disease well at home even though they know how to do it and are willing to do it.

**Trial registration:**

National Library of Medicine, with ClinicalTrials.gov, Identifier: NCT05456906. https://clinicaltrials.gov/ct2/show/NCT05456906.

## Introduction

Theoretically, hospitalizations or emergency department (ED) visits due to ambulatory care-sensitive conditions (ACSCs), such as hypo-/ hyperglycemia, heart failure, and asthma, are potentially avoidable if patients can receive effective primary care and follow the medical regimen in the community (1, 2). However, ACSC hospitalizations and ED visits are still costly, even in modern countries. For example, in 2009, Great Britain spent £1.4 billion on ACSC hospitalizations (3). In 2016, the United States spent about $1.7 billion on treating ACSC ED visits for Medicare fee-for-services beneficiaries (4), about $33.7 billion on ACSC hospitalizations for adults, and $ 0.5 billion on ACSC hospitalizations for children in 2017 (5). Nevertheless, socially vulnerable patients consume the majority of ACSC expenses.

Billings and colleagues published their findings regarding the association between socioeconomic status and ACSC hospitalizations based on the data in New York City in 1993 (6). After that, literature has accumulated rich quantitative evidence about a persistent discrepancy in ACSC hospitalizations or ED visits due to social risk factors (e.g., deprivation and race/ethnicity) from time to time (7–11). There is a growing interest in using patients' narratives or stories to improve the quality of health care and policies (12) because patients' narratives through qualitative studies provide rich information about how they manage their diseases in the community, which are hard to be detected by quantitative approaches.

The present study aims to meta-synthesize evidence from qualitative studies focusing on social risk factors that contribute to ACSC hospitalizations or ED visits. In the end, the present study provides implications for policymakers and stakeholders to address social determinants of health upstream through policies and implications for healthcare practitioners to assist patients at the downstream level to reduce costly ACSC hospitalizations and ED visits. The present study also provides guidance for future research.

## Materials and methods

The Agency for Healthcare Research and Quality (AHRQ) defined the conditions of ACSC in the Preventive Quality Indicator Program. These ACSCs included diabetes-related acute and chronic complications, heart failure, hypertension, chronic obstructive pulmonary disease, asthma, uncontrolled diabetes, and low-extremity amputation due to diabetes (1). The present study reviewed the qualitative studies that focused on hospitalizations or ED visits due to ACSCs for adults. We chose hospitalizations and ED visits because about 40% of ACSC ED visits were referred for hospitalizations (4).

### Design

The study is a meta-synthesis study following the procedural steps that were proposed by Noblit and Hare (13) and developed by Sandelowski and Barroso (14), to create analytical themes regarding the social risk factors that contribute to ACSC hospitalizations or ED visits. The Preferred Reporting Items for Systematic Reviews and Meta-Analysis were strongly recommended in studying systematic review and meta-analysis (15), which was used to reach the objective of the present study. We thematically synthesized the evidence related to adult patients who experienced any of the ACSC hospitalizations or ED visits.

### Eligible criteria

The inclusion criteria included primary peer-reviewed qualitative studies that focused on adult patients experiencing ACSC hospitalizations or ED visits and were published in English-language journals, with no restriction on publication date or country of origin. Only qualitative evidence was included for the studies based on mixed methods. We focused on patients' experiences and narratives; therefore, the perspectives of parents, caregivers, and healthcare professionals were excluded. Studies were also excluded if they specifically focused on readmissions, preventable conditions not defined by AHRQ (e.g., cellulitis or chest pain), patients in institutions, and patients with dementia, cancer, or mental illness.

### Search strategies

We systematically searched PubMed, Embase, Cochrane Library, and Web of Science databases to identify all qualitative studies investigating ACSC. At the beginning, we did not set the beginning of the year for literature searching in order to maximize the pool for the qualitative studies. After an initial search, we decided to set the time frame of the literature search from 2013 to June 2022, as it was determined that there had been limited qualitative research on ACSC prior to this date. Articles were retrieved in English, and the publication types were limited to primary research reports. The following search terms were used as free text in the field of title/abstract: (ACSC) OR (ambulatory care sensitive conditions) OR (ambulatory care-sensitive conditions) OR (avoidable hospitalization) OR (preventable hospitalization) OR (avoidable emergency) OR (preventable emergency), and in the ALL Field: (qualitative) OR (narrative) OR (observation) OR (mixed method) OR (mixed-method) OR (interview) OR (focus group) OR (case study). One author (HC) independently screened the title and abstract to exclude quantitative studies. After that, two authors (HC and HL) independently reviewed abstracts and full texts for eligibility of qualitative studies and studies based on mixed methods. The full texts of the articles were examined in terms of inclusion criteria in cases where the title or the abstract was not sufficient. Following by Mu' suggestion (16), the two authors independently evaluated the studies and made a co-decision in case of disagreement or doubt. A consensus was reached through the discussion between the two authors. When the two authors had different opinions on the review of studies, a third consultant expert was asked to resolve the decision.

### Quality appraisal

The Critical Appraisal Skills Programme (CASP) provides a series of checklists for different types of research, such as randomized control trials, cohort studies, and qualitative studies. The present study used the checklist for qualitative studies, which had been commonly used to evaluate the quality of qualitative studies (17, 18). The CASP checklist has 10 questions, including (1) a clear statement of the aim of the study; (2) the appropriateness of the methodology; (3) the appropriateness of the study design; (4) the appropriateness of the recruitment strategies; (5) the appropriateness of data collection; (6) taking the relationship between researchers and patients into consideration; (7) ethical concerns; (8) rigor of the data analyses; (9) a clear statement of findings, and (10) the value of the findings (19). Two authors (HC and HL) independently used the checklist to evaluate the quality of the qualitative studies before synthesis and reached a consensus through the discussion. Table 1 presents the evaluation results.

**Table 1:** Evaluation of the quality of the studies.

**Checklist**	**Quensell**	**Sentell**	**Shearer**	**Granger**	**Manski-N**.	**James**	**Ridge**	**Pasciak**	**Longman**
A clear statement of the aim of the study	Y	Y	Y	Y	Y	Y	Y	Y	Y
The appropriateness of the methodology	Y	Y	Y	Y	Y	Y	Y	Y	Y
The appropriateness of the study design	Y	Y	Y	Y	Y	Y	Y	Y	Y
The appropriateness of the recruitment strategies	Y	Y	Y	Y	Y	Y	Y	Y	Y
The appropriateness of data collection	Y	Y	Y	Y	Y	Y	Y	Y	Y
Taking the relationship between researchers and patients into consideration	C	C	C	C	C	Y	Y	C	C
Ethical concerns	Y	Y	Y	Y	Y	Y	Y	Y	Y
Rigor of the data analyses	Y	Y	Y	Y	Y	Y	Y	Y	Y
A clear statement of findings	Y	Y	Y	Y	Y	Y	Y	Y	Y
The value of the findings	Y	Y	Y	Y	Y	Y	Y	Y	Y

Y, Yes; C, Cannot tell.

CASP provides “Hint” for each question to help researchers make the choices. The sixth questions “Has the relationship between researcher and participants been adequately considered?” The Hint for the “relationship” question includes: (1) “If the researcher critically examined their own role, potential bias and influence during (a) formulation of the research questions (b) data collection, including sample recruitment and choice of location,” and (2) “How the researcher responded to events during the study and whether they considered the implications of any changes in the research design.”

We selected “Cannot Tell” when the study did not clearly describe all of the above criteria.

### Data abstraction

All potential articles were entered into Excel and a bibliographic software program (Mendeley, developed by Elsevier, Mendeley Ltd.). The two authors reviewed the articles based on the inclusion and exclusion criteria from the title/abstract. The full text was evaluated if the abstract did not provide sufficient information. The cited articles listed in the reference of the articles were also reviewed.

### Meta-synthesis

Thematic content analysis steps suggested by Braun and Clarke (20) were followed in synthesizing the findings of qualitative research included in the study. In the first stage, all research articles were read by two authors (HC and HL), and a code was given to each basic finding using NVivo-version-11-software. In the second stage, these codes were compared with the studies, the similarities and differences between the codes were examined and transformed into descriptive themes (risk factors for ACSC hospitalizations/ ED visits). In the third stage, analytical themes (core theme, major themes, and subthemes) were developed by discussing the descriptive themes and study purpose to obtain a synthesis product that could put forward new concerns, problems, or suggestions in light of the research subject with an inductive approach. The themes and subthemes must be exclusive to each other. The two authors (HC and HL) investigated the similarities and differences in findings across studies and the meanings of patients' narratives and then synthesized findings and patients' narratives into each theme. The two authors (HC and HL) also reviewed and revised the narratives themes, and subthemes until reaching an agreement. When the two authors had different opinions on the data synthesizing, a third consultant expert was asked to resolve the findings.

## Results

**Figure 1:**
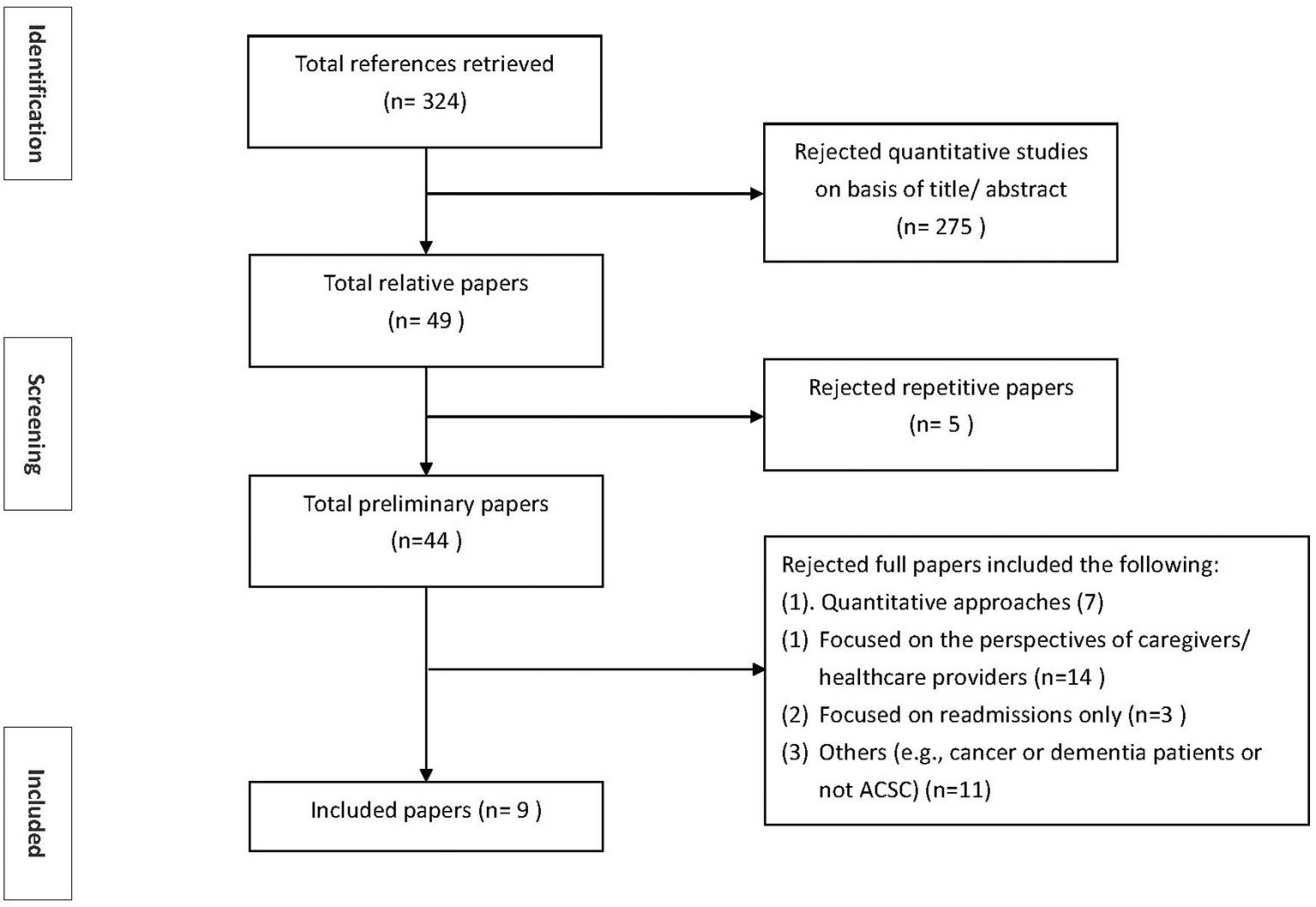
Flowchart of the meta-synthesis steps.

Following the criteria, search strategies, study selection, and data extraction discussed above, we initially retrieved 324 references. We excluded 275 quantitative articles based on the title or abstract. We further excluded 5 articles due to duplications. Additionally, we excluded the articles because they were quantitative (7 articles) or focused on caregivers and health care professionals (14 articles), readmissions only (3 articles), and others (e.g., not explicitly focused on ACSCs or focused on cancer patients) (11). The number of qualitative articles included in the present study was nine. One of the nine studies written by Qunesell and colleagues specifically focused on housing issues from 21 patients who were a part of 90 patients in the study of Sentell et al. (21, 22). Figure 1 presents the selection process for the study sample.

The qualitative data in the nine studies were primarily based on semi-structured in-person or telephone interviews at hospitals, ED, clinics, or patients' residences. The total unique number of patients in the nine studies was 167, ranging from 3 to 90 patients per study. All studies investigated ACSC hospitalizations except one that studied ACSC ED visits (23). Two studies used a mixed-method study design (24, 25). The data analyses were primarily content or thematic analyses.

Although the Agency for Healthcare Research and Quality grouped ACSC into chronic conditions (e.g., diabetes and heart failure) and acute conditions (i.e., bacterial pneumonia and urinary tract infection) (1), all these nine qualitative studies investigated patients with chronic conditions. In addition, three studies focused on one specific disease (e.g., diabetes or heart failure) and others studied more than one chronic ACSC condition.

### Critical appraisal skills programme assessment

Table 1 shows the quality of qualitative studies based on the 10 questions in the CASP checklist. Nine studies (21–29) in the present study meet all criteria except for one—taking the relationship between researchers and patients into consideration. All studies described the characteristics of interviewers, analysts, and researchers; however, only two studies had a clear statement to explain the roles of interviewers to patients (26, 27). Although we could not evaluate “taking the relationship between researchers and patients into consideration” for the other seven studies, based on the CASP criteria and the information provided by the selected studies, the quality of the seven studies remained since the research questions, interview guidelines, and interviewers were defined before the authors conducted the studies. Based on the suggestion by Mu (16), we included all of the nine studies in this meta-synthesis study.

### Descriptive findings of the study sample and patient characteristics

Table 2 presents the descriptive findings of the study sample. A total of 167 non-duplicated adult patients were included in the nine studies (excluding one child from James' study and 21 patients from Quensell's study because they were a part of the study sample in Sentell's study in Table 2). The minimal age of patients was 18 years old. The majority of the patients were middle-aged and older. Four studies provided the race/ethnicity of their patients (22, 23, 27, 28), while other studies discussed patients' income or poor neighborhoods (21–23), indicating that patients were socially vulnerable. Two studies did not provide a gender distribution of patients (23, 26). The nine studies were conducted in two countries: four in Australia and five in the United States.

**Table 2:** Descriptive findings of the study samples.

**References**	**Sampled/ disease**	**Hospital/ ED visits**	**N**	**Race/ethnicity**	**Age (Yrs)/ gender/ income**	**Data collection technique**	**Data analysis (technique; rigor)**	**Location or country**
Quensell et al. (21)^a^	Diabetes and heart failure	Hosp.	21	Native Hawaiian/ Pacific Islander: 55.6%; White: 18.9%; Asian: 13.3%, Filipino: 12.2%	18–64 yrs: 71%; 65+ yrs: 28.9%; female: 33%; family income < $40,000: 57.8%; depression 52.2%	Face-to-face interview for about 45 min; a questionnaire and semi-structured interview	Framework approaches, consensus	Queen's Medical Center, Hawaii, U.S.
Sentell et al. (22)	Diabetes and heart failure	Hosp.	90	Native Hawaiian/ Pacific Islander: 55.6%; White: 18.9%; Asian: 13.3%, Filipino: 12.2%	18–64 yrs: 71%; 65+ yrs: 28.9%; female: 33%; family income < $40,000: 57.8%;	Semi-structured interview.	Framework approaches, consensus	Queen's Medical Center, Hawaii, U.S.
Shearer et al. (23)	African Americans with Type 2 DM	ED visits	20	African Americans	30–88 yrs; household income $800-$8,000 per month	Semi-structured interview at patients' home	Explanatory framework	Southeastern state (South Carolina), US
Granger et al. (24)^b^	New York Association class II-IV heart failure.	Hosp.	10	NA	48–81 yrs (mean 67 yrs). 50% male	Open-ended questions for about 30–75 min	Meaning-response interview and thematic	Duke Hospital, US
Manski-Nankervis et al. (25)^b^	Type 2 diabetes experiencing angina or AMI or foot ulceration	Hosp.	13	NA	22–87 Yrs (mean: 64 yrs) with 53.8% female	Semi-structured interview, ranging for 30 min to 1 h	Thematic framework	Royal Melbourne Hospital and Werribee Mercy Hospital in Australia.
James et al. (26)^c^	Type 1 diabetes, with symptoms of diabetic ketoacidosis	Hosp.	4/18	NA	(3 adults, 1 parent of a child, and 18 health workers)^b^	Semi-structured face-to-face or telephone interview	Gibbs's Thematic framework, Consensus	Caboolture, Australia— Socio-economic disadvantage area
Ridge et al. (27)	Rural patients experienced ACSC hospitalizations	Hosp.	10	NA	47–91 yrs (mean: 68) male: 6 and female: 4	Semi-structured telephone interviews (21–37 min., mean 29 min.)	Reflexive thematic analysis	Tasmania, Australia
Pasciak et al. (28)	Diabetes patients experiencing hypoglycemia	Hosp.	17	African American: 64.7%; white: 35.3%	≥65 (mean: 78.9 yrs), female: 76.5%,	In-person on-on-one in-depth open-ended interview	Constant comparative method, thematic emergence, and consensus	Yale-New Haven Hospital and Saint Raphael Hospital, U.S.
Longman et al. (29)	COPD, CHF, angina or diabetes complication	Hosp.	24	NA	52–88: < 70 yrs: 50%; 70+yrs: 50% male:14 female: 10	Semi-structured, one-to-one telephone interviews after discharge from hospital (median days: 15, ranging from 13 to 38 min)	Thematic analysis	Two regional hospitals in Australia

^*a*^The 21 patients are part of the 90 patients in the Sentell's study.

^*b*^The studies were mixed method. The present study only included qualitative evidence from patients who participated in the qualitative study.

^*c*^The present study only included the findings from 3 adult patients.

### Descriptive themes: core theme and four major themes

**Figure 2:**
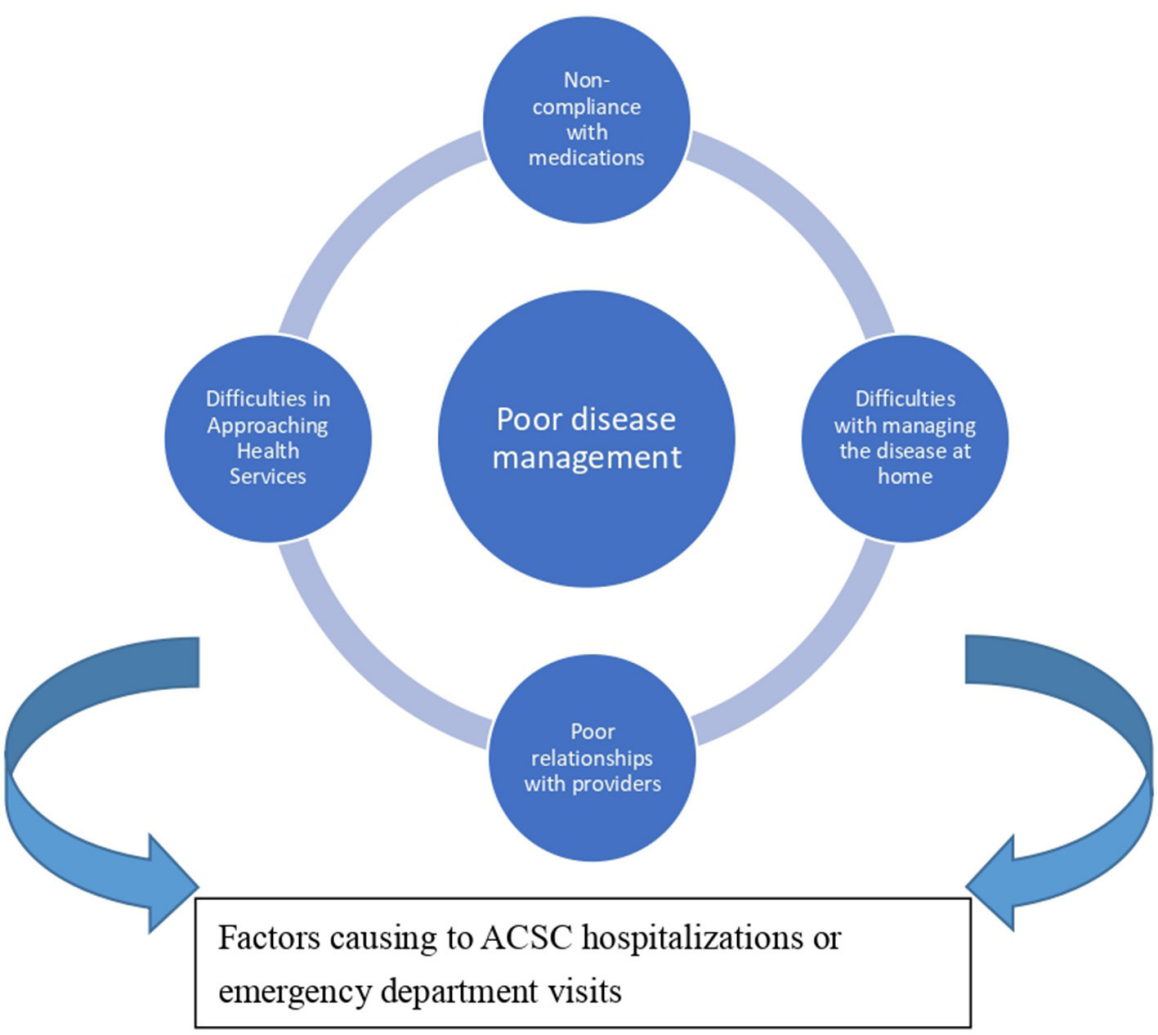
Core theme and major themes.

Based on the meta-synthesized analysis, a framework regarding the themes for causing ACSC hospitalizations or ED visits from patients' narratives is developed (Figure 2). The core theme is poor disease management, which is defined as the central mechanism describing why socially vulnerable patients were unable to manage their disease well, thereby causing ACSC hospitalizations or ED visits. We also identified four major themes leading to poor disease management. Table 3 presents four major themes, including difficulty in approaching health services, non-compliance with medications, difficulties in managing the disease at home, and poor relationships with providers. Each major theme contains 2–4 subthemes. To describe the weight of each subtheme, the number of citations and the percentage (the number of citations for a theme divided by the total number of studies) were also presented in Table 3. The detailed citations, their corresponding subtheme, and major theme were displayed in Supplement Table 1, which allows readers to examine the data collected and analyzed by the authors, to understand the findings of the analysis, and to evaluate the plausibility, credibility or face validity of the authors' claims (30, 31).

**Table 3:** Descriptive themes: ACSC hospitalizations/ emergency department visits from patient perspectives.

**Theme**	**Sub-themes (Risk Factors)**	**Citations with experience**		
		***n***	**%**	**Reference No**
**Core theme: Poor disease management**				
1.Difficulties in approaching health service	(1)Lack of insurance	2	22.2	(22, 23)
	(2)Lack of mobility assistance	5	55.6	(21–23, 26, 27)
	(3)Financial constraints	6	66.7	(21–23, 25, 26, 29)
	(4)Inaccessible health care	6	66.7	(22, 23, 25–27, 29)
2. Non-compliance with medications	(1)Low health literacy	6	66.7	(22–24, 27–29)
	(2)Psychosocial or cognitive constraints	6	66.7	(21–23, 25, 27, 29)
	(3)Conflicting demand	4	44.4	(22, 25, 28, 29)
	(4)Unwillingness	4	44.4	(22, 23, 26, 29)
3.Difficulties with managing the disease at home	(1)Unstable housing	2	22.2	(21, 22)
	(2)Lack of family and social support	5	55.6	(22, 23, 27–29)
4.Poor relationship with providers	(1)Perception of incompetent providers	3	33.3	(22, 26, 29)
	(2)Poor communication and coordination	7	77.8	(21–23, 25–28)
	(3)Language/cultural barriers	1	11.1	(22)

We individually discussed each major theme and the corresponding subthemes and patient narratives below.

#### Theme 1: difficulties in approaching health services

Theme 1 included four subthemes: (1) lack of health insurance, (2) lack of mobility assistance (e.g., transportation), (3) financial constraints, and (4) inaccessible health care. Among these subthemes, the most cited are financial constraints and inaccessible health care (66.7%); the second most cited was lack of mobility assistance (55.6%) while lack of health insurance was cited twice (22.2%). The examples of financial constraints included no money to pay the copay of medications (22, 23, 29) and to purchase healthy food and testing suppliers for diabetes control (22, 25, 26). The examples of inaccessible health care included difficulty in getting an appointment (23, 25) and no ambulatory services after-hour clinics (22, 27). Lack of transportation for physician appointments or refilling medication was also often cited (21–23, 26, 27).

#### Theme 2: non-compliance with medications

Theme 2 comprised four subthemes: low health literacy, psychosocial or cognitive constraints, conflicting demand, and unwillingness. Among these subthemes, low health literacy and psychosocial or cognitive constraints had 66.7% cited by selected studies. For example, four studies noted that patients did not know their disease, symptoms, or warning signs (22, 23, 28, 29) and three found that patients did not know how to apply dietary control or disease management in daily life (22, 27, 29). Patients also had various psychosocial or cognitive constraints, such as stress or depression worsening health status (22, 29), emotional distress on disease management (23, 25), or neglecting to refill or take medication (22, 23, 29). About 44.4% of the studies cited conflicting demand and unwillingness, such as low priorities for disease management (22, 25) and unfeasible planned care at home (22).

#### Theme 3: difficulties with managing the disease at home

Lack of family and social support and unstable housing are subthemes leading to difficulties with managing the disease at home. 55.6% of the selected studies noted family and social support issues, such as living alone or having no one to rely on when necessary (22, 23, 27–29). Although several narratives were about housing issues, such as no running water for medication or no refrigerator to store insulin (21, 22), they were primarily from two studies because one of the two studies mainly focused on housing issues.

#### Theme 4: poor relationships with providers

There were three subthemes: perception of incompetent providers (33.3%), poor communication and coordination (77.8%), and language/ cultural barriers (11.1%). Several narratives, such as no communication and coordination between physicians, pharmacists, and specialists (22, 26, 27) and lack of trust (21, 22) highlight the issue of poor communication and coordination under the major theme. Also, the perception of the incompetence of providers, such as culture and language barriers, leads to poor relationships with patients.

## Discussion

### Summary of the key findings

There were 167 non-duplicated patients from the studies conducted in the United States and Australia. Those patients had at least one chronic disease and faced significant obstacles to managing their chronic diseases properly in the community. The core theme identified in the present study was poor disease management, which comprised four major themes: difficulties in approaching health services, noncompliance with medications, difficulties in managing the disease at home, poor relationships with providers. Under each major theme, there are 2–4 subthemes, including lack of insurance, lack of mobility assistance, financial constraints, inaccessible health care, unstable housing, lack of family and social support, low health literacy, psychosocial and cognitive constraints, conflicting demand, unwillingness, perception of incompetent providers, poor communications and coordination among providers, and language and culture barriers. It is worth noting that among all subthemes, poor communication and coordination was most cited by the selected studies (77.8%). The second most cited were financial constraints, inaccessible health care, low health literacy, psychosocial or cognitive constraints, lack of mobility assistance, and lack of family and social supports.

### Contributions of the study

The present study is unique in two ways. First, to the best of our knowledge, the present study is the first to conduct a meta-synthesized analysis based on qualitative evidence regarding ACSC hospitalizations or ED visits. Second, differing from previous quantitative studies that quantified the association between social risk factors and ACSC hospitalizations or ED visits, our findings revealed that poor disease management, the core theme of the study, plays the central role that turns socially vulnerable patients at risk of ACSCs hospitalizations or ED visits.

### Policy implications

Our findings show that upstream social determinants of health, such as financial constraints, low health literacy, lack of family and social support, contribute to poor disease management and major themes, leading to ACSC hospitalizations or ED visits in the present study. These upstream social determinants are beyond what healthcare professionals can manage. The United States spent about $34 billion on ACSC hospitalizations in 2017 (5). Suppose policymakers or stakeholders are willing to use half of the expense ($17 billion) as the budget to mitigate the effect of the poor upstream social determinants pf health through social or health policies, socially vulnerable individuals can gain control of their disease management. In the long run, the return on investments is expected to be high. For example, education is associated with health literacy (32, 33). In our findings, several narratives indicated low health literacy of the patients, such as not knowing the disease, symptoms, warning signs, or how to apply dietary control or disease management in the daily life (22, 23, 27–29), which led to non-compliance with medications. Education is effective and has a long-term effect on health, income, and employment, especially for children and women (34, 35). Although the return on investment for education may take years, policies that enhance education typically early childhood education, would have a life-long impact on individual and population health.

Additionally, reducing financial barriers through health policies is essential for individuals with chronic diseases to manage their disease.

Although Australia has universal health insurance coverage and most of the participants in Sentell et al. study had insurance coverage through the Medicaid program in the United States, high copays for medications motivated patients not to take or refill medications (22, 23). Costly medical supplies (e.g., ketone testing strips) also prevented patients from periodically monitoring their blood sugar (26). Our findings are consistent with previous evidence, indicating that uninsured patients likely skipped required healthcare services (36, 37), which risked their life and was eventually costly to society. Nevertheless, some medications and medical supplies are life-saving. For example, insulin or an insulin pen is required for insulin-dependent patients to prevent short-term complications (e.g., coma due to hyperglycemia) and long-term complications (e.g., renal failure or blindness) or early death. In 2020, the RAND Corporation indicated that the average price of insulin in the United States was $98.7 per vial, much higher than in other countries (38). Health policies at the state level that make life-saving medications and medical supplies affordable are strongly recommended.

### Implications to clinics at the provider's level and payment policy

#### Assessment of social risk factors and interventions at clinics

Assessing patients' social risk factors is as critical as assessing patients' health problems during patient-healthcare practitioners' encounters at the clinics. Patients included in the selected studies are vulnerable due to their socioeconomic status and race/ethnicity. Based on our findings, these patients had weak social support systems and limited resources to navigate the healthcare system, as well as distrusted healthcare providers and did not communicate well with providers, which is consistent with previous findings (39, 40). These vulnerable patients are high-cost patients to society. To enhance patients' compliance with disease management, healthcare professionals and other professionals (e.g., social workers) need to work together to identify vulnerable patients and tailor medical and social services based on individual needs to make sure that personalized medical regimen is applicable and manageable to patients in the community.

#### Assessment of social risk factors for payment policies

Payment adjustment based on patients' social risk factors is commonly discussed and recommended (41). Without accounting for patients' social risk factors, payment is unfair and likely widens disparities in care. A good example is the Hospital Readmissions Reduction Program (HRRP) in 2013 in the United States. The 2013 HRRP was to reduce 30-day readmissions while improving the quality of care for Medicare beneficiaries. The 2013 HRRP financially penalized hospitals with 30-day readmissions higher than the national average, without considering patients' social risk factors. As a result, the 2013 HRRP financially penalized safety-net hospitals that took care of the poor and minorities and further weakened the financial abilities of safety-net hospitals (42–45).

The Centers for Medicare and Medicaid Services Center (CMS) and the American Academy of Family Physicians in the United States have published social needs screening tools. The CMS Accountable Health Communities Health-Related Social Needs (HRSN) Screening Tool is for healthcare providers in clinics or hospitals to collect social needs data for Medicare and Medicaid beneficiaries (46). The HRSN tool includes 26 questions that cover social risk factors in 13 domains: housing instability, food insecurity, transportation problems, utility help needs, interpersonal safety, financial strain, employment, family and community support, education, physical activity, substance use, mental health, and disabilities (46). The American Academy of Family Physicians published the Social Needs Screening Tool under the EveryONE Project in 2018 (47). The screening tool includes 15 questions that cover 10 dimensions: housing, food, transportation, utilities, childcare, employment, education, finance, personal safety, and assistance. It is recommended to integrate different social needs screening tools in order to simplify and ease the workload for health professionals at clinics or hospitals. Furthermore, given our findings, each social risk factor likely plays a different weight in poor disease management. Therefore, the payment adjustment based on a rigorous methodology that appropriately weights individual social risk factors would likely motivate healthcare providers to take care of socially vulnerable patients and further reduce disparities in health outcomes.

#### Implications to future research

Although ACSC hospitalizations or ED visits have been studied for decades, to the best of our search from the database, we only identified nine qualified qualitative studies published in English-language journals. These studies were conducted in the United States and Australia. Different countries have different contexts and systems, which may affect ACSC hospitalizations or ED visits differently. More qualitative studies from different countries would provide a better understanding of the mechanisms regarding how social risk factors lead to costly ACSC hospitalizations and ED visits. Additionally, our study only focused on adult patients. Future studies investigating ACSC hospitalizations or ED visits from the perspectives of children and their caregivers and the perspectives of healthcare providers are suggested. Furthermore, among the nine studies, only one study focused on ED visits. About 40% of ED visits were referred to inpatient care (4). Patients discharged from ED without hospitalizations may differ from those admitted to hospitals for inpatient care. Investigating patients with ACSC ED visits would help to provide a better understanding of the differences in associated social risk factors between ACSC hospitalizations and ACS ED visits. Finally, AHRQs grouped ACSCs into acute and chronic conditions. Our study sample primarily focused on chronic ACSCs; therefore, qualitative studies focusing on acute ACSCs (i.e., bacterial pneumonia and urinary tract infections) are also recommended.

### Limitations

There are a few limitations in the present study. Socially vulnerable patients are likely to be minorities and have low socioeconomic status. Among the nine qualitative studies in our study sample, five studies did not report the race/ethnicity of the participants, and six studies did not report the income or education of the participants. For future qualitative studies, it is recommended to provide race/ethnicity, income, education, and employment status to describe the characteristics of the participants. Furthermore, the selected qualitative studies were primarily from Australia and the United States. The generalizability of the findings from the presented studies may not be able to apply to other countries because every country has different healthcare delivery systems and social contexts.

## Conclusions

There is a growing interest in using patients' narratives or stories to improve the quality of health care and policies. Our present study is the first to apply a meta-synthesis approach to investigate ACSC hospitalizations or ED visits based on qualitative evidence. Our findings showed that poor disease management is the core theme that turns socially vulnerable individuals with chronic disease at risk of ACSC hospitalizations or ED visits. Although modern medical science has improved disease management through innovative medicine and technology (e.g., insulin for diabetes control and diuretic and beta-blocker for heart failure), medical science alone is unlikely to reduce costly ACSC hospitalizations or ED visits, especially for socially vulnerable individuals. In order to reduce costly ACSC hospitalizations or ED visits among socially vulnerable patients, addressing upstream social determinants of health is fundamental.

## Data availability statement

The original contributions presented in the study are included in the article/Supplementary material, further inquiries can be directed to the corresponding author.

## Author contributions

HFC conceptualized the study, obtained funding, designed the study, conducted quality check, led the coding and analysis of the qualitative data and theme identification, and wrote the first draft of the manuscript. HRL conceptualized the study, designed the study, conducted quality check, led the coding and analysis of the qualitative data and theme identification and provided critical feedback and edited the manuscript. All authors have read and approved the final version of the manuscript.

## References

[1] Agency for Healthcare Research and Quality. Prevention quality indicators technical specifications updates-Version V2020 (ICD 10-CM/PCS) (2020). Available online at: https://qualityindicators.ahrq.gov/archive/pqi_techspec/icd10_v2020 (accessed May 11, 2018).

[2] Bindman AB Grumbach K Osmond D Komaromy M Vranizan K Lurie N . Preventable hospitalizations and access to health care. JAMA. (1995) 274:305–11. 10.1001/jama.1995.03530040033037 7609259

[3] Tian Y Dixon A Gao H. Data briefing-emergency hospital admissions for ambulatory care-sensitive conditions: identifying the potential for reductions. King's Fund (2012). Available online at: http://www.kingsfund.org.uk/sites/files/kf/field/field_publication_file/data-briefing-emergency-hospital-admissions-for-ambulatory-care-sensitive-conditions-apr-2012.pdf (accessed August 10, 2022).

[4] Lesser A Israni J Lo AX Ko KJ. Older adult visits to the emergency department for ambulatory care sensitive conditions. J Am Coll Emerg Physicians Open. (2020) 1:824–28. 10.1002/emp2.12164 33145526 PMC7593478

[5] McDermott KW Jiang HJ. Characteristics Costs of Potentially Preventable Inpatient Stays, 2017: Statistical Brief #259. (2020). Available online at: https://www.hcup-us.ahrq.gov/reports/statbriefs/sb259-Potentially-Preventable-Hospitalizations-2017.jsp (accessed July 25, 2022).

[6] Billings J Zeitel L Lukomnik J Carey TS Blank AE Newman L. Impact of socioeconomic status on hospital use in New York City. Health Aff. (1993) 12:162–73. 10.1377/hlthaff.12.1.162 8509018

[7] Davis SK Liu Y Gibbons GH. Disparities in trends of hospitalization for potentially preventable chronic conditions among African Americans during the 1990s: implications and benchmarks. Am J Public Health. (2003) 93:447–55. 10.2105/AJPH.93.3.447 12604494 PMC1447762

[8] Hale N Probst J Robertson A. Rural area deprivation and hospitalizations among children for ambulatory care sensitive conditions. J Community Health. (2016) 41:451–60. 10.1007/s10900-015-0113-2 26516019

[9] Mukamel DB Ladd H Li Y Temkin-Greener H Ngo-Metzger Q. Have racial disparities in ambulatory care sensitive admissions: abated over time? Med Care. (2015) 53:931–9. 10.1097/MLR.0000000000000426 26421373 PMC4607653

[10] McCormick D Hanchate AD Lasser KE Manze MG Lin M Chu C . Effect of massachusetts healthcare reform on racial and ethnic disparities in admissions to hospital for ambulatory care sensitive conditions: retrospective analysis of hospital episode statistics. BMJ. (2015) 350:h1480. 10.1136/bmj.h1480 25833157 PMC4382709

[11] Maxwell J Cortes DE Schneider KL Graves A Rosman B. Massachusetts' health care reform increased access to care for Hispanics, but disparities remain. Health Aff. (2011) 30:1451–60. 10.1377/hlthaff.2011.0347 21821562

[12] Grob R Schlesinger M Barre LR Bardach N Lagu T Shaller D . What words convey: the potential for patient narratives to inform quality improvement. Milbank Q. (2019) 97:176–227. 10.1111/1468-0009.12374 30883954 PMC6422610

[13] Noblit GW Hare RD. Meta-ethnography: Synthesizing Qualitative Studies. Sage: Newbury Park (1988). 10.4135/9781412985000

[14] Sandelowski M Barroso J. Handbook for Synthesizing Qualitative Research. Springer: New York (2007).

[15] Page MJ Moher D Bossuyt PM Boutron I Hoffmann TC Mulrow CD . PRISMA 2020 explanation and elaboration: updated guidance and exemplars for reporting systematic reviews. BMJ. (2021) 372:n160. 10.1136/bmj.n160 33781993 PMC8005925

[16] Mu PF. Qualitative systematic review research method. Yuan-Yuan Nursing. (2014) 8:5–11.

[17] Campbell R Pound P Pope C Britten N Roisin P Morgan M . Evaluating meta-ethnography: a synthesis of qualitative research on lay experiences of diabetes and diabetes care. Soc Sci Med. (2003) 56:671–84. 10.1016/S0277-9536(02)00064-3 12560003

[18] Rozbroj T Haas R O'Connor D Carter SM McCaffery K Thomas R . How do people understand overtesting and overdiagnosis? Systematic review and meta-synthesis of qualitative research. Soc Sci Med. (2021) 285:114255. 10.1016/j.socscimed.2021.114255 34391966

[19] CASP Critical Appraisal Skills Programme. CASP qualitative checklist. CASP Online (2018). Available online at: https://casp-uk.net/casp-tools-checklists/ (accessed July 3, 2022).

[20] Braun V Clarke V. Using thematic analysis in psychology. Qual Res Psychol. (2006) 3:77–101. 10.1191/1478088706qp063oa

[21] Quensell ML Taira DA Seto TB Braun KL Sentell TL. I need my own place to get better: patient perspectives on the role of housing in potentially preventable hospitalizations. J Health Care Poor Underserved. (2017) 28:784–97. 10.1353/hpu.2017.0074 28529224 PMC5630224

[22] Sentell TL Seto TB Young MM Vawer M Quensell ML Braun KL . Pathways to potentially preventable hospitalizations for diabetes and heart failure: a qualitative analysis of patient perspectives. BMC Health Serv Res. (2016) 16:300. 10.1186/s12913-016-1511-6 27456233 PMC4960879

[23] Shearer JE Jenkins CH Magwood GS Pope CA. Contested ownership of disease and ambulatory: sensitive emergency department visits for type 2 diabetes. Am J Med Sci. (2016) 351:400–6. 10.1016/j.amjms.2016.01.007 27079346 PMC4836170

[24] Granger BB McBroom K Bosworth HB Hernande A Ekman I. The meanings associated with medicines in heart failure patients. Eur J Cardiovascu Nurs. (2013) 12:276–83. 10.1177/1474515112447734 22653088

[25] Manski-Nankervis A Furler J Audehm R Blackberry I Young D. Potentially preventable hospitalisations: are they a useful marker of access to and experience of care in general practice among people with type 2 diabetes? Aust J Prim Health. (2015) 21:214–20. 10.1071/PY13112 24491142

[26] James S Annetts K Frakking T Broadbent M Waugh J Perry L . Diabetic ketoacidosis presentations in a low socio-economic area: are services suitable? BMC Health Serv Res. (2021) 21:682. 10.1186/s12913-021-06715-7 34246266 PMC8272902

[27] Ridge A Peterson GM Seidel BM Vinah Anderson V Nash R. Rural patients' perceptions of their potentially preventable hospitalisation: a qualitative study. J Patient Exp. (2022) 9:23743735211069825. 10.1177/23743735211069825 35005222 PMC8733360

[28] Pasciak WE Berg DN Cherlin E Fried T Lipska KJ. Qualitative analysis of reasons for hospitalization for severe hypoglycemia among older adults with diabetes. BMC Geriatr. (2021) 21:318. 10.1186/s12877-021-02268-w 34001014 PMC8130109

[29] Longman JM Rix E Johnston JJ Passey ME. Ambulatory care sensitive chronic conditions: what can we learn from patients about the role of primary health care in preventing admissions? Aust J Prim Health. (2018) 24:304–10. 10.1071/PY17191 30078392

[30] Lincoln YS Guba EG. Naturalistic Inquiry. Sage: Thousand Oaks (1985). 10.1016/0147-1767(85)90062-8

[31] Lin HR. Qualitative analysis, discussion and rigor; In Nursing Research and Practice. Princeton: Taipei (2020). p. 349–64.

[32] Bundy DAP de Silva N Horton N Jamison DT Patton GC. Disease Control Priorities, Third Edition (Volume 8): Child Adolescent Health Development. Washington, DC: World Bank. © World Bank (2017). Available online at: https://openknowledge.worldbank.org/handle/10986/28876

[33] Pradhan E Suzuki EM Martínez S Schäferhoff M Dean TJ Jamison DT. The effects of education euantity and euality on child and adult mortality: their magnitude and their value. In: Child and Adolescent Health and Development: Disease control priorities. 3rd Ed. (Vol 8): The World Bank (2017). p. 423–40. 10.1596/978-1-4648-0423-6_ch30 30212146

[34] OECD. Health at a glance 2021: OECD indicators. Paris: OECD Publishing (2021).

[35] Wilper AP Woolhandler S Lasser KE McCormick D Bor DH Himmelstein DU . national study of chronic disease prevalence and access to care in uninsured U. S adults. Ann Intern Med. (2008) 149:170–6. 10.7326/0003-4819-149-3-200808050-00006 18678844

[36] Mulcahy A Schwam D Edenfield N. Comparing insulin prices in the United States to other countries: Results from a price index analysis (2020). Available online at: https://www.rand.org/content/dam/rand/pubs/research_reports/RRA700/RRA788-1/RAND_RRA788-1.pdf (accessed July 25, 2022).

[37] Hayes SL Salzberg CA McCarthy D Radley DC Abrams MK Shah T . High-need, high-cost patients: who are they and how do they use health care? A population-based comparison of demographics, health care use, and expenditures. Issue Brief. (2016) 26:1–14. 27571599

[38] Salzberg CA Hayes SL McCarthy D Radley DC Abrams MK Shah T . Health system performance for the high-need patient: a look at access to care and patient care experiences. Issue Brief. (2016) 27:1–12. 27571600

[39] Office of the Assistant Secretary for Planning Evaluation. Report to congress: Social risk factors and performance in medicare. (2020). Available online at: https://aspe.hhs.gov/sites/default/files/migrated_legacy_files//195046/Social-Risk-in-Medicare's-VBP-2nd-Report-Executive-Summary.pdf (accessed August 20, 2022).

[40] Joynt KE Jha KA. Characterisitcs of hospitals receiving penalities under the hospital readmissions reduction program. JAMA. (2013) 309:342–3. 10.1001/jama.2012.94856 23340629

[41] Hu J Gonsahn MD Nerenz DR. Socioeconomic status and readmissions: evidence from an urban teaching hospital. Health Aff. (2014) 33:778–85. 10.1377/hlthaff.2013.0816 24799574

[42] Karim SA Nevola A Morris ME Tilford JM Chen HF. Financial performance of hospitals in the Appalachian region under the hospital readmissions reduction program and hospital value-based purchasing program. J Rural Health. (2021) 37:296–307. 10.1111/jrh.12475 32613645

[43] Chen HF Karim S Wan F Nevola A Morris ME Bird TM . Financial performance of hospitals in the Mississippi Delta region under the hospital readmissions reduction program and hospital value-based purchasing program. Med Care. (2017) 55:924–30. 10.1097/MLR.0000000000000808 29028756

[44] The Centers for Medicare Medicaid Services. The Accountable Health Communities Health-Related Social Needs Screening Tool. (2017). Available online at: https://innovation.cms.gov/files/worksheets/ahcm-screeningtool.pdf (accessed October 1, 2022).

[45] O'Gurek DT Henke C. A practical approach to screening for social determinants of health. Fam Pract Manag. (2018) 25:7–12. 29989777

[46] Sadowski LS Kee RA VanderWeele TJ Buchanan D. Effect of a housing and case management program on emergency department visits and hospitalizations among chronically ill homeless adults. JAMA. (2009) 301:1771–8. 10.1001/jama.2009.561 19417194

[47] UN Habitat: for a better urban future. Housing (2022). Available online at: https://unhabitat.org/topic/housing (accessed August 11, 2022).

