# Supplementary material for: Social determinants of ambulatory care sensitive conditions: a qualitative meta-synthesis based on patient perspectives
Source: Front Public Health. 2023 May 9;11:1147732. doi: 10.3389/fpubh.2023.1147732 (PMC10203230; doi:10.3389/fpubh.2023.1147732)
Supplement: Supplementary file 1 [file Table_1.doc]

Supplement Table 1: Themes, subthemes, and Corresponding Citations from Patient Perspectives

| **Core theme: Poor Disease Management** | | |
| --- | --- | --- |
| **Theme** | **Subthemes** | **Citations** |
| Difficulties in approaching health service | Lack of insurance (22,23) | - No insurance to visit physicians (22,23) - No medical insurance to refill medications (22,23) - Did not take medications for heart disease for a long time because of no insurance (22) |
|  | Lack of mobility assistance (21,22,23,26,27) | - Lack of transportation for physician appointments (21,22,23,26,27) - Miss an appointment due to a broken elevator (21) - Lack of transportation for refilling medications (21,22,23,26,27) |
|  | Financial constraints (21,22,23,25,26,29) | - No money to buy food but medications needs to be taken with food (22) - No money afford copay for medications (22,23,29) - Costly ketone testing suppliers (26) - Costly diabetes control, from eating healthy to exercise to blood testing (22,25) - Services are unaffordable (29) - Financial responsibility to other family members (21) |
|  | Inaccessible health care (22,23,25,26,27,29) | - Unavailability of ambulatory services after-hour clinics (22,27) - Lack of ketone testing supplies in local stores (26) - Lack of home care or therapists (29) - Waiting for specialists (25,29) - Could not get in touch with physicians (22) - Hard to get an appointment or seeing a different doctor each time (23,25) - Beyond the scope of general practitioners (27) |
| Non-compliance with medications | Low health literacy (22,23,24,27,28,29) | - Do not know what drugs are (22,24) - Too many medications three times a day or too many medications to manage (22,24) - Did not know their diseases, symptoms, or warning signs (22,23,28,29) - Did not know how to apply dietary control or disease management in daily life (22,27,29) - Perception of misdiagnosis from hospitals (29) - Lack of preventability / misconceptions about triggers of health problems (29) - Did not know that morning blood sugar is needed when taking medication (22) |
|  | Psychosocial or cognitive constraints (21,22,23,25,27,29) | - Embarrassing to visit doctor due to not quitting smoking (29) - Don’t want go to hospital because of worry about home affairs (29) - Stopping taking medications because of losing wife, job, home and taking drug (22) - Forgetting or neglecting to take medications (23,29) - Reluctant to take medications (23) - Fear of medication side-effects (29) - Neglect to refill medication (22,23) - Not taking medications at the right time or dose (22) - Stress or anxiety / depression worsening health status (22,29) - Feel discriminated due to disability (21) - A constant emotional burden for having diabetes (23) - Self blame and emotional distress on disease management (23,25) - Complexity and confusion medication regimens due to multi-comorbidity (29) - Only visit general practitioners when sick (27) |
|  | Conflicting demand (22,25,28,29) | - Managing health as the second priority due to lots of responsibilities (22) - Managing diabetes is at the low priority due to other issues (25) - Struggle with balancing a diet from prevent hyperglycemia and hypoglycemia at the same time (28) - Hard to balance between not gaining weight and avoiding hypoglycemia (28) - No exercise due to physical constraints (sore legs) (29) - Tension between clinician-prescribed treatment and self-management for patients with polypharmacy or taking insulin (28) - The planned care did not work at home (e.g., do not walk but chores at home requires walk) (22) |
|  | Unwillingness (22,23,26,29) | - The denial of having disease (22) - Want to enjoy life and avoid the significant lifestyle change (e.g., exercise or diet, not quitting smoking for COPD) for disease management (22) - Do not care anymore or resign to fate (22,23,29) - Wait until the health conditions became too bad (26) |
| Difficulties with managing the disease at home | Unstable housing (21,22) | - Ignore health problems or not visiting physicians due to feeling shame of being homeless (21) - Unable to take medications because of no running water due to homeless (22) - Lack a refrigerator to store insulin (21) - Unable to prepare their food for diabetes control due to lack of electricity in the apartment (21) - Eat high-salty canned food at shelters or churches due to homeless (21) - Overdrink due to no air condition in the apartment in hot summer for heart failure patients (21) - Lack clean water to prevent wound infection (21) - Unable to schedule time to take or manage medications due to homeless (21) - Unable to access medication due to eviction (21) - Limited access to fresh products and healthy food (21,22) - Feel stigma and discriminated in daily life and during the interactions with health care providers due to homeless (21) |
|  | Lack of family and social support (22,23,27,28,29) | - No one provides advice or to rely on when necessary (22,23,27,29) - Live alone and unable to seek for help (28) |
| Poor relationships with providers | Perception of incompetent providers (22,26,29) | - Healthcare professionals were not aware of condition change and down played with patients’ report and experiences (26) - Physicians do not understand patients’ culture (22) - Premature discharge from hospitals (22,29) - Received inadequate care from previous hospitalizations (22) |
|  | Poor communication and coordination (21,22,23,25,26,27,28) | - Patients with diabetes and heart disease did not tell physicians about their depression or drinking behaviors (22) - Experienced poor communication with providers (e.g., Providers did not take time with patients, used complex words, and did not have local and culture knowledge (22) - Not respect the privacy of patients (22,23) - Lack of trust between patient and health care provider (21,22) - Do not listen to patients (25)Poor communication and coordination among healthcare providers (e.g., it seems physicians, pharmacists, and specialists did not talk to each other) (22,26,27) - Guided by general physicians or phone line to seek care at ER (23,27) - Lack of Follow-up services (after surgery) (27) - Lack physicians who look after patients for a long run (25) - Medication was not delivered to patients’ house (28) |
|  | Language/ cultural barriers (22) | - Patients did not understand the languages and feel shame to ask (22) |
